# Supplementary material for: Mucin Transiently Sustains Coronavirus Infectivity through Heterogenous Changes in Phase Morphology of Evaporating Aerosol
Source: Viruses. 2022 Aug 24;14(9):1856. doi: 10.3390/v14091856 (PMC9503081; doi:10.3390/v14091856)
Supplement: Supplementary file 1 [file viruses-14-01856-s001.zip › viruses-1776487-supplementary.pdf]

Supplementary Materials

Table S1. Measurements from Figure 2. MHV is an appropriate surrogate for SARS-CoV-2

	RH %	pH	Time (seconds)	N	Infectivity (%)
Figure 2a Aerosol phase infectivity	90		30	6	M=86.50, SD=41.85
	90		120	10	M=68.79, SD=34
	90		300	10	M=67.02, SD=11.84
Figure 2b Aerosol phase infectivity	40		30	11	M=42.78, SD=15.54
	40		120	6	M=41.70, SD=15.36
	40		300	7	M=38.64, SD=14.64
Figure 2c Bulk liquid infectivity		7.5	15	15	M=100, SD=13.83
		7.5	600	12	M=117.01, SD=41.74
		8.0	15	4	M=100, SD=47.75
		8.0	600	4	M=91.96, SD=11.60
		8.5	15	11	M=100, SD=13.62
		8.5	600	15	M=83.04, SD=23.81
		9.5	15	12	M=100, SD=39.04
		9.5	600	8	M=67.35, SD=23.81
		10.0	15	15	M=100, SD=29.47
		10.0	600	11	M=40.11, SD=15.63
Figure 2d Bulk liquid infectivity		8.5	15	14	M=100, SD=13.84
		8.5	120	9	M=80.95, SD=11.85
		8.5	300	12	M=82.00, SD=15.18
		8.5	600	15	M=83.04, SD=22.38
		9.5	15	14	M=100, SD=35.9
		9.5	120	9	M=86.72, SD=18.32
		9.5	300	12	M=73.17, SD=23.1
		9.5	600	8	M=67.35, SD=8.42
		10.0	15	15	M=100, SD=29.47
		10.0	120	3	M=61.35, SD=5.24
		10.0	300	12	M=34.99, SD=15.66
		10.0	600	11	M=40.11, SD=15.63

Table S2. Measurements from Figure 3. Comparing MHV infectivity in two droplet sizes.

	Initial droplet radius (µm)	Time (seconds)	N	Infectivity %
Figure 3b Aerosol phase infectivity	25 ±1	30	6	M=52.65, SD=17.7
		120	3	M=39.14, SD=3.59
		300	6	M=46.58, SD=6.23
	31 ±1	30	11	M=42.79, SD=15.54
		120	6	M=41.77, SD15.37
		300	7	M=38.64, SD=14.64

Table S3. Measurements from Figure 4. Effect of mucin on airborne MHV infectivity with two tailed t-test

	Time (seconds)	Mucin concentration w/v (%)	N	Infectivity %	*t*	*p*
Figure 4a Aerosol phase infectivity	30	0	*11*	*M* = 42.78 *SD* = 15.54		
		0.1	*13*	*M* = 64.79 *SD* = 20.50	19 = 2.42	.02
		0.3	*6*	*M* = 57.11 *SD* = 9.44	15 = 2.04	.05
		0.5	*5*	*M* = 77.37 *SD* = 19.88	15 = 3.98	.001
Figure 4b Aerosol phase in-fectivity	120	0	*6*	*M* = 41.70 *SD* = 15.39		
		0.1	*6*	*M* = 55.31 *SD* = 20.79	11 = 1.31	.21
		0.3	*11*	*M* = 58.22 *SD* = 13.63	10 = 1.96	.07
		0.5	*7*	*M* =68.03 *SD* = 27.76	15 = 2.14	.05
Figure 4c Aerosol phase in-fectivity	300	0	*7*	*M* = 38.64 *SD* = 14.64		
		0.1	*9*	*M* = 46.65 *SD* = 16.68	13 = 0.98	.34
		0.3	*8*	*M* = 36.75 *SD* = 19.50	10 = 0.19	.85
		0.5	*8*	*M* = 45.16 *SD* = 15.92	12 = 0.79	.44

Table S4. Standardised distribution of T=0 measurements

Standardised distribution of T=0 descriptive statistics	
Mean	100.82
Standard Error	3.58
Median	101.37
Mode	119.04
Standard Deviation	27.93
Sample Variance	780.10
Kurtosis	-0.41
Skewness	-0.18
Range	127.20
Minimum	32.05
Maximum	159.25
Sum	6150.15
Count	61

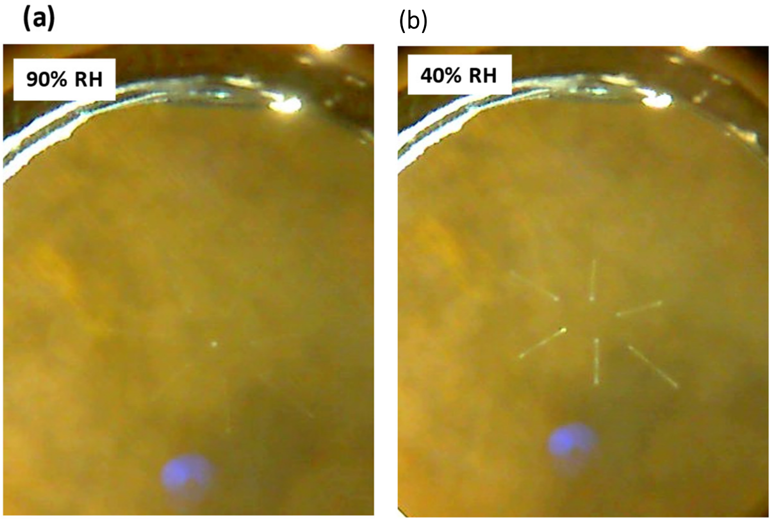

Figure S1. Qualitative evidence for efflorescence. A planar view of the CELEBS chamber, visualising levitated aerosols after 30 seconds. (a) RH 90% (b) RH 40%. Aerosol particles appeared to reflect the light at a higher intensity at 40% RH, appearing brighter in chamber view, when compared with equilibrated droplets levitating at 90% RH. This is indicative of the aerosol particles at 40% RH containing a solid crystalline component and scattering the light irregularly with more intensity than the liquid bioaerosol at 90% RH.

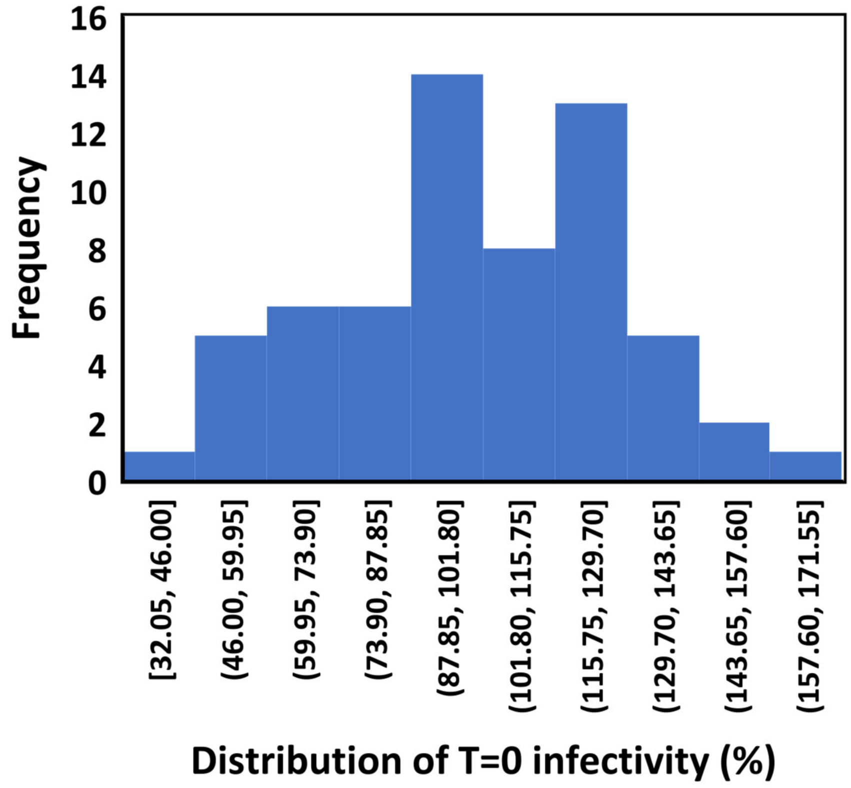

Figure S2. Standardised distribution of T=0. Levitations (5 seconds) at 90% RH were shown to result in no observable loss of infectivity compared with the initial viral titre measured through $TCID_{50}$. The data from each separate measurement requires standardisation to an average of the initial viral load carried by a droplet at the beginning of the levitation, referred to as the T=0 measurement from that day.

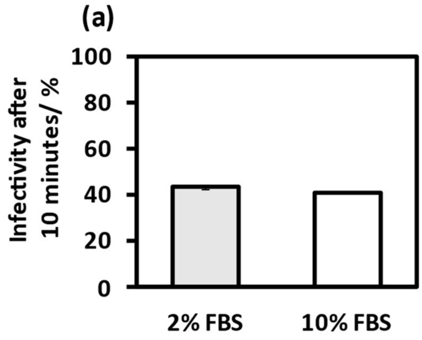

Figure S3. Bulk liquid viral infectivity at high pH, comparing two concentrations of FBS. Mean viral infectivity in bulk phase after 10 minutes at pH 9.8.

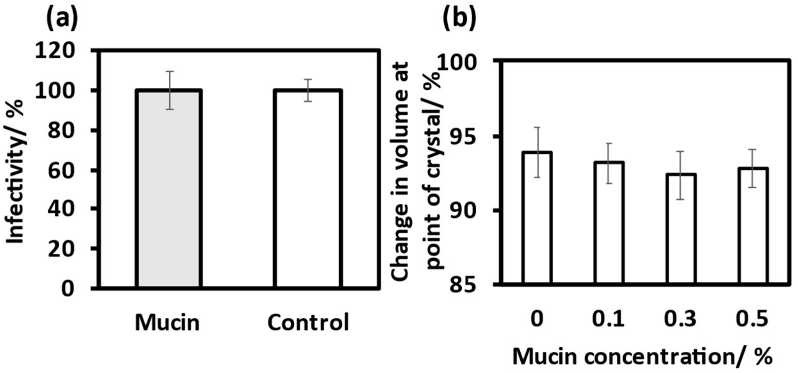

Figure S4. Effect of mucin on bulk phase infectivity and evaporation kinetics. (a) Mean infectivity of virus in bulk phase with 0.5% w/v mucin, and 0% w/v mucin control, error bars show standard error. (B) Percentage change in volume of evaporating droplet with mucin concentration, measured using CK-EDB, error bars show standard deviation (N=100).

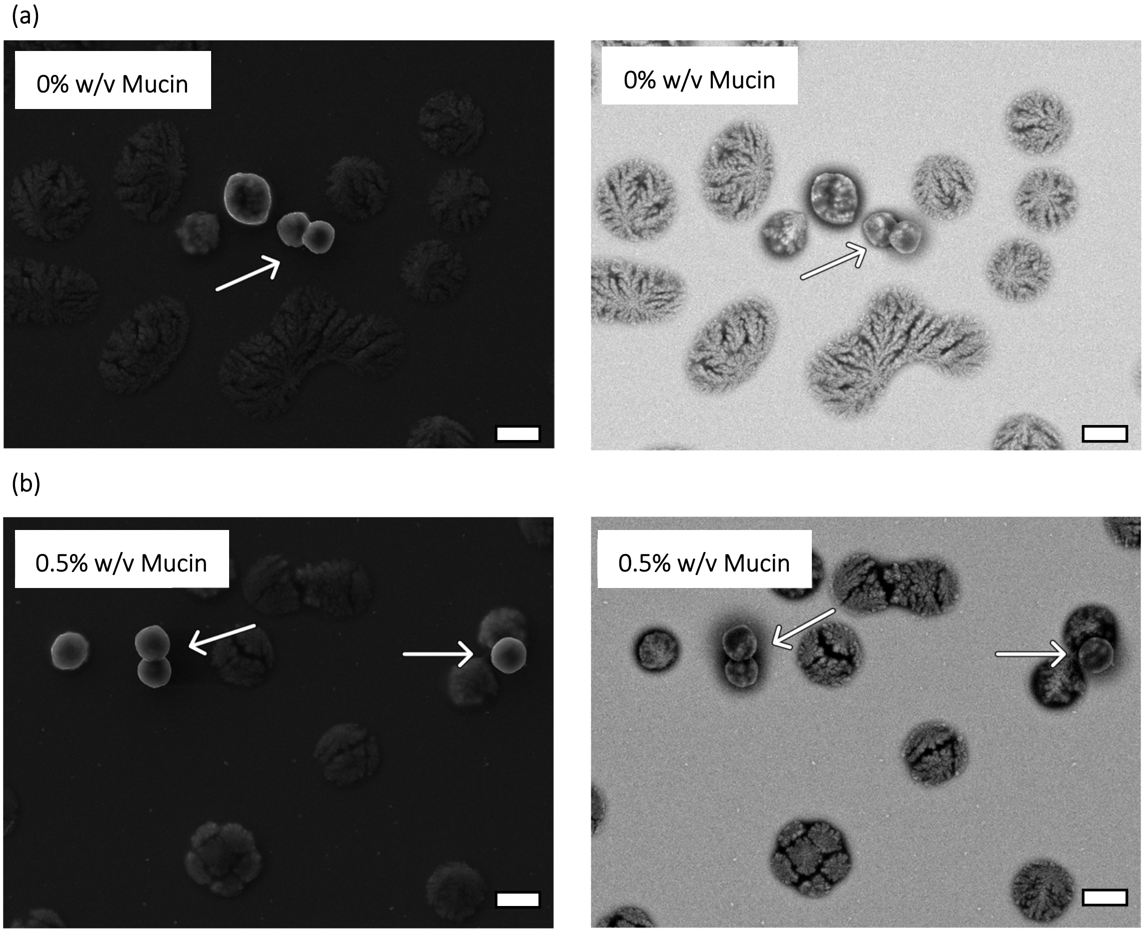

Figure S5. SEM micrographs of particles collected from the bottom of FDC. Droplets dried at 40% RH. Magnification x1000, scale bar 5 μm. Particles indicated by white arrows. SED images show topographical structure, left column. BED-C images show internal phase separation, right column (a) DMEM 10% FBS 0% mucin (b) DMEM, 10% FBS, 0.5% w/v mucin.
